# Mouth Breathing

**Published:** 1886-04

**Authors:** C. H. Bushong

**Affiliations:** 337 West 50th Street


### MOUTH-BREATHING.

C. H. BUSHONG, M. D.

" 'Mouth-breathing,' said a distinguished physician, 'is a curious affliction. It is more common than is supposed, occurs in infancy, at manhood, and at middle age, and causes a good deal of suffering; yet it is a strange thing that the people in general do not seem to pay any attention to this affliction when occurring in their own families, in spite of the manner in which it distorts the face, until some actual disease sets in and medical aid is necessary. Even the physicians do not seem to reflect that this trouble may cause any one of a large number of diseases affecting the system in general. Why, you can tell one of these mouth-breathers anywhere the moment you see him. From disease of the nose, his lips are retracted, his mouth is continually open, his gums recede, and his teeth protrude, particularly those in the upper jaw, the flesh that forms the lower part of the nostrils is shrunken, the openings of the nostrils are diminished in size, there are wrinkles at the outer edge of the eyes, and deep lines run from the nostrils to the angles of the mouth. These all give the person either an expression of idiocy, silliness or suffering. But the principal thing is the neccessity of parents and nurses watching children when they fall asleep, and preventing them from breathing with their mouths open. In grown persons, diseases of the nose and throat may lead to mouth-breathing and the resulting distortions of the features; but it is the other way with children. They should be taught to use the mouth for eating and speaking only; and if they fall asleep with their mouths open the lipsshould be gently pressed together. Thus, many constitutional diseases, such as spinal trouble, pigeon breast, and perhaps even rickets, may be avoided, not to speak of the affections of the throat and lungs.'

It seems that medical men are not the only ones who have studied more or less carefully the habit of mouth-breathing. George Catlin, the portrayer of American Indian life and customs, claimed in one of his works that it was a known fact that a man can inhale mephitic air through the nose for a certain time in the bottom of a well without harm; but, if he opens his mouth to answer a question or call for help, his lungs are closed, and he expires. Catlin says:—

'I have seen a poor Indian woman in the wilderness lowering her baby from her breast and pressing its lips together as it falls asleep.'

Among two million people, he found that deafness, dumbness, spinal curvature, and deaths from teething and diseases of the respiratory passages were almost unknown. He attributes this exemption from these ailments, so very common in civilized life, solely to the habit of breathing through the nose."

The above was handed to me by the editor of Hall's Journal of Health. It came from a subscriber who writes that he is a sufferer from this condition, and asks if anything can be done to help him.

The physician quoted has mentioned the symptoms of this "affliction," as he truly calls it. The sender of the article complains of another and a very common one: It is the result of sleeping with the mouth open, and is noticed on awaking. During the time of sleep the membranes of the mouth and throat have been exposed to the evaporating influences of air in favorable conditions for taking up moisture,—warm and in motion. These mucous membranes are not provided with the facilities for keeping up the supply of secretion, nor have they the extent of surface coming in contact with this moving air with which the proper channel for it is provided. Consequently we are not surprised when he complains of "soreness in the throat,' "dry rough tongue," has a bad taste in his mouth and feels dull and unrefreshed after his sleep. We should not be surprised, either, if his wife (?) should complain of his *snoring*, as it is one of the results of sleeping with the mouth open.

These unpleasant conditions of mouth and throat are soon followed by a chronic inflammation of the mucous membrane of these parts, making "catarrh" a natural sequela of mouth-breathing. This with its accompanying discomforts, is bad, yet worse is in store for him if he continues the practice.

Nature provided the nose as a breathing apparatus, and it is admirably adapted to its purpose. By its passage through the somewhat tortuous naral canal, the cold air is moderated, and the more delicate interior of the lungs are protected from its chilling effects. In this manner much bodily heat is saved, for this inspired air if cold would take warmth directly from the blood. This small amount saved by proper breathing is taken off eighteen to twenty times each minute if we breath through the mouth. Very little calculation will show how great a drain is thus made on the resources of the body to keep the temperature up to the normal.

But this not all. The mucous membrane of the nose is lined with *ciliated epithelium*,—that is, cells with tiny hairs. These little hairs (ciliæ) act as a sieve and catch many small masses of dust, pollen of plants, etc., that are always in the air, and as they—the ciliae—are continually waving toward the outlet they are thus capable of removing these foreign bodies. When the air is taken in through the mouth it does not come in contact with any of these protectors of the lungs until it reaches the larynx and trachea. These are large tubes, and only a limited portion of the air comes in contact with their surface. The consequence is that many flakes of dust and various injurious things get into the lung. Thus air that comes to the lungs through the mouth has less warmth, less moisture, and more dirt than that taken in through the nose. The lung is chilled by it, the blood loses heat and moisture and the floating bodies find a lodgement and frequently become foci of disease processes that often culminate in permanent or fatal lung trouble.

When we think of treatment it is first important that we understand mouth-breathing to be a *habit*. An inherited form of teeth or lips may cause a tendency towards its acquirement, yet that it is a habit is evidenced by many cases in which it has been overcome merely by persistent effort. With infants its earliest evidences should be checked, as in the example of the Indian mother. The formation of the habit is thus avoided, and the child saved from a long train of injurious effects in the future. There is no danger of smothering, as the child will wake if the nose is obstructed. Mothers and nurses should see that these little noses are kept free, and they can save their charges from this unsightly affliction if they are only watchful.

With mature mouth-breathers much of the treatment rests with themselves. They must first have a physician or surgeon to remove any causes that interfere with the free passage of air through the nose. These may exist in the form of "catarrh," polypi, distorted septum, etc., all of which are curable. After this has been done the patient must do the rest. The younger he begins, the better his chances are for a complete recovery, because less perseverance will be required. Many will say that they cannot keep their mouths closed while rsleep. This is true, but if they persist and breathe only through the nose during the time they are awake, they will find that their mouths will remain closed when they sleep. It must be remembered that no habit can be cured in a short time. The most constant watchfulness will be required for months, and perhaps for years, but it will ultimately be rewarded by a cure.

A recent paper contains an advertisement of a "Mouth-breathing Inhibitor," to be worn at night to avoid the unpleasant dryness, etc., that result from sleeping with the mouth open, and also to prevent snoring. I never have seen one of these, but I see no reason why it should not be of benefit. The main danger is that any one using any means to aid in *curing* a habit is only pandering to his own lack of pluck. He looks to means outside of himself to do that which he must do by his own force of will and self-control. To seek external aids for conquering habits is a sign of weakness, and a yielding to it only increases his helplessness. He who would be free must arouse himself and be a man, self-reliant and self-controlling.

"There's the marble, there's the chisel,
Take it, work it to thy will;
Thou alone can shape thy future,
Heaven send thee strength and skill."

337 West 50th Street.

